# A Treatise on the Science and Practice of Midwifery

**Published:** 1893-12

**Authors:** 


A TREATISE ON THE SCIENCE AND PRACTICE OF MIDWIFERY. By W. S. Playfair, M. D.., F. R. C. P., Professor of Obstetric Medicine in King's College, London; Examiner in Midwifery to the Universities of Cambridge and London, and to the Royal College of Physicians. Sixth American from the eighth English edition. Edited with additions, by Robert P. Harris, M. D. In one octavo volume of 697 pages, with 217 engravings and 5 plates. Cloth, $4.00; leather $5.00. Philadelphia, Lea Brothers & Co., 1893.

PLAYFAIR'S SYSTEM OF MIDWIFERY: HARRIS.—A very successful attempt has been made to employ in this work all recent advancement in the science and art of Midwifery. The author, though bringing it fully abreast with British advancement has fallen short of the ideas entertained on this side of the Atlantic. For this, however, there is compensation in the notes by American editor.

In the body of the work, the author presents in a clear and condensed manner the subjects treated, avoiding much unnecessary reading matter so irksome to the average medical student.

There are a few faults, an example of which is found in the chapter treating of abortions; more emphasis should have been placed upon the necessity of antiseptics and the early and complete evacuation of the uterus, rather than tolerating a delay of a "day or two at most."

Again, after pains are regarded as "salutary in effect" while we look upon them as a danger signal.

Parts conflicting with the opinions expressed in the notes by the American editor would have been better omitted, as it is better for the student to first learn the correct and latest thing than to plod through ideas exploded, to unlearn them in the end. Notably is this the case in caesarean section and rupture of the uterus. This, however, is but a blemish in the construction of the book as the most recent advancements are embodied in the notes by Dr Harris.

This volume compares very favorably with others and is better than many that we have seen.

As an epitome it is as full as it could be without going beyond its purpose.

For the "student and busy practitioner" it is happily arranged, and is written in an easy and pleasant style.

